# Supplementary material for: RNA-mediated symmetry breaking enables singular olfactory receptor choice
Source: Nature. 2023 Dec 20;625(7993):181–8. doi: 10.1038/s41586-023-06845-4 (PMC10765522; doi:10.1038/s41586-023-06845-4)
Supplement: Supplementary file 1 — Supplementary Notes and Fig. 1. [file 41586_2023_6845_MOESM1_ESM.pdf]

nature portfolio

https://doi.org/10.1038/s41586-023-06845-4

Supplementary information

RNA-mediated symmetry breaking enables singular olfactory receptor choice

In the format provided by the authors and unedited

Supplemental Note 1

Data Analysis & Calculations

Multiome Experiments
Data processing with Signac
Signac 1.6.0 and Seurat 4.1.0 developed by the Satija Lab was used for all analyses which was performed in R 4.1.2.

Data input and quality control
All data was loaded and processed following the vignette described by the Satija Lab online (https://stuartlab.org/signac/articles/pbmc_multiomic.html). Briefly, outputs from *cellranger-arc count,* filtered_feature_bc_matrix.h5 (RNA) and atac_fragments.tsv.gz (ATAC), were loaded and combined into a Seurat object, and Chromatin Assay, respectively. The multiome was quality controlled by retaining cells with a TSS enrichment > 1, nucleosome signal < 2, ATAC counts between 1,000 and 10,000, and RNA counts between 1,000 and 25,000. Peaks were called using macs2 v2.2.7.1 on the entire multiome, using the fragment file as the input to the peak calling algorithm, CallPeaks, with the following arguments: -g 2.7e9 -f BED –nomodel –extsize 200 –shift -100, and removing peaks overlapping annotated genomic blacklist regions for the mm10 genome.

Gene expression and DNA accessibility processing, and cell annotation
Gene expression UMI count data was normalized using SCTransform and we performed PCA using the RunPCA function in Seurat. Dimension reduction was performed on the "peaks" Chromatin Assay using LSI, with the following functions FindTopFeatures (arguments: min.cutoff = 5), RunTFIDF, and RunSVD. Joint UMAP was performed using the FindMultiModalNeighbors function to calculate closest neighbors using a weighted combination of RNA and ATAC similarities. Clusters were identified using weighted nearest neighbors data and were annotated by their expression of known marker genes for cells in the olfactory neuronal lineage [57,58].

mOSN cCRE Identification
mOSN marker genes were identified by using the FindMarkers function on RNA data from the mOSN cluster. Only genes detected in > 25% of cells were extracted. To identify cCREs of top mOSN marker genes we performed peak-to-gene linkage using the LinkPeaks function with the following arguments: peak.assay = "peaks", expression.assay = "SCT", distance = 5e+05, min.distance = 2000, min.cells = 10, n_sample = 200, pvalue_cutoff = 0.05, score_cutoff = 0.2.

scATAC tile plots & percent accessibility per enhancer
The TilePlot function was used to generate tile plots of the number of fragments within a window surrounding Greek Islands and a cCRE. The FeatureMatrix function was used to extract fragments within a bed file of Greek Islands and mOSN cCREs. The percent accessibility per enhancer was measured by calculating the frequency with an enhancer had at least one fragment (Tn5 insertion event) across all mOSNs.

Pseudotime projection with Monocle3 & verification with expression of neuronal marker genes
We converted our MOE multiome Seurat object to a CellDataSet using the *as.cell_data_set* function from monocle3 1.0.0, and projected it into low-dimensional space via UMAP. We ordered cells and chose the beginning-most node in the GBC cluster as the root node for trajectory generation. We then verified the accuracy of the trajectory by examining the SCT normalized expression of known marker genes: GBC – *Ascl1*, INP1/2 – *Neurod1*, INP3 – *Tex15*, iOSN – *Gap43*, and mOSN – *Carns1*. For each gene the mean and standard error of expression were calculated per pseudotime, then both the mean and standard error were scaled according to the maximum average expression of the gene across all pseudotimes, to allow for superimposed comparison of all marker genes on the same scale. We called this "scaled mean and standard error" of gene expression.

Genome accessibility and max OR transcript levels over pseudotime
The FeatureMatrix function was used to extract fragment counts within bed files of four genomic groups: Greek Islands, OR promoters, mOSN cCREs, and Lhx2 and EBF1 bound peaks in mOSNs (identified in Monahan et al. 2017 [12]), and were normalized to the total number of ATAC counts per cell. Cumulative accessibility per

genomic group per cell was extracted by finding the sum total number of normalized fragments per enhancer across all loci within a genomic group, which was then averaged for all cells sharing the same pseudotime rounded to the nearest integer. To measure the accessibility of only active GIs, we measured the average normalized fragment count of only active GIs per cell, defined as having at least 1 Tn5 insertion event, which was then plotted across pseudotime.

Greek Island Accessibility Patterning and OR choice
The mOSN cell cluster was extracted from the MOE Seurat object. 1,010 unique mOSNs were permuted pairwise to generate 509,545 unique combinations. For each combination, the number of accessible Greek Islands shared between the two cells was measured. Cell pairs were binned by their number of common Greek Islands to calculate the frequency of cell pairs possessing between 0 (minimum) to 12 (maximum) Greek Islands in common (Fig. 1L). A cell's chosen OR was assigned by identifying the single most highly expressed OR. To calculate OR frequencies across populations of cell pairs with varying degrees of overlap in accessible GIs, first all unique cells were extracted in each bin of *n* GIs in common per cell pair. The frequency of each OR expressed among all unique cells per bin was averaged to determine the average frequency of an OR among all cells in a bin, which is plotted as a dot (mean) with error bars (standard error). The red dashed line represents the predicted OR frequency if OR choice was "random" and not linked to GI constitution, where OR frequency = 1/number of unique cells per bin (Fig. 1M). Cells that did not have any accessible GIs, or that did not express a single OR higher than all others were excluded from the analysis. For mOSNs in Extended Data Fig. 1, only wildtype cells were examined by excluding P2+ cells.

Dip-C Experiments
Greek Island Hub Patterning
36 mor28iGFP nuclei and 42 *gg8tTA>tetOP2iGFP* nuclei were compared between themselves and each other for overlap in the combination of *trans* GIs within 2.5 p.r. of the active OR and therefore in the active GIH (mor28-vs-mor28, n = 630 cell pairs; P2-vs-P2, n = 861 cell pairs; mor28-vs-P2, n = 1453 cell pairs). GIs from chr7 and chr14 are excluded from this analysis to examine GIs that are *trans* to both P2 and mor28. Cell pairs were binned by their number of common Greek Islands to calculate the frequency of cell pairs possessing between 0 (minimum) to 3 (maximum) Greek Islands in common.

Hierarchical clustering of Greek Island spatial relationships
Refer to Extended Data Fig. 2A for schematic. To identify active and inactive enhancer complexes, conda 4.11.0 was used to create a virtual environment with Python v2.7.13, where the function 'dip-c pd' from the dip-c package (https://github.com/tanlongzhi/dip-c) was called to generate symmetric matrices, measured in particle radii (p.r.), of the pairwise distance relationships between all Greek Islands as well as the active OR allele. We scaled distance matrices and performed hierarchical clustering based off the euclidean relationships between all elements in a matrix using the hclust function from the R package "dendextend." We cut dendrograms at a height of 2.5 p.r. using the cutree function, to extract all branches with an average radius of the same distance, which was verified in Extended Data Fig. 6d.

Identification of Active GIH, Inactive GIHs, Inactive GIH most similar to Active GIH, and OR genes within GIHs
The active GIH was identified as the branch containing the active OR allele by hierarchical clustering (see above). For CSS comparison of active and inactive hubs, only the inactive GIH in every cell that had the most similar concentration of GIs as the active GIH in the same cell was used for CSS analysis. This inactive GIH was identified by counting the number of GIs in a cell's active GIH, and finding another GIH (dendrogram branch) containing the most similar number of GIs (Extended Data Fig. 6a). The similar size and density of active GIH and the most similar inactive GIH was verified in Extended Data Fig. 6d-e. OR genes contained within GIHs were identified by using the dip-c pd function to extract all ORs within 5 p.r. of all GIs participating in a GIH.

Contact specificity score in Dip-C
Haplotype imputed single-cell contacts were extracted as described in https://github.com/tanlongzhi/dip-c, binned at 50-kb resolution, and normalized to per cell library size. To compute single-cell CSS, contacts within a 1-Mb radius surrounding two regions of interest were measured, for all regions of interest in a cell, and summed across cells, generating a single 2-Mb-by-2-Mb matrix of contacts. Contact specificity scores were generated by normalizing the contacts within each 50-kb-by-50-kb bin to the total number of contacts in the matrix. The

normalized contacts in the 50-kb-by-50-kb bin at the center of the matrix was measured to generate the CSS for the interaction of interest. Only interchromosomal contacts were analyzed.

PCA on Publicly Available Dip-C Data
Single-cell chromatin compartments were generated exactly as described in Tan et al. 2019 using MOE cells from Tan et al. 2019. Briefly, single-cell chromatin compartment for each 1-Mb bin was calculated as the average CpG frequency of all other bins contacting it, weighted by the number of contacts, with 'dip-c color2' (with parameters '-b1000000 -H -c color/mm10.cpg.1m.txt'), and rank-normalized to 0–1 in each cell (requires Bedtools). Bulk Hi-C libraries were downsampled to 25M reads, aligned in the dip-c pipeline and chromatin compartments of bulk Hi-C data were calculated in the same way as in single cells (namely, with 'dip-c color2'). Chromatin compartments of each bulk sample (a vector of compartment values for all 1-Mb bins along the genome) were then projected onto the PCA space generated from single cells.

Bulk Hi-C and Liquid Hi-C Experiments
In situ Hi-C, Liquid Hi-C, and HiChIP contact analysis & contact specificity score calculation (CSS)
Contacts were extracted at 50-kb resolution from cooler files and normalized to contacts per billion for comparison between libraries. To compute CSS, contacts within a 1-Mb radius surrounding two regions of interest were computed, between all regions of interest in one condition and summed, generating a 2-Mb-by-2-Mb matrix of contacts. Contact specificity scores were generated by normalizing the contacts within each 50-kb-by-50-kb bin to the total number of contacts in the matrix. The normalized contacts in the 50-kb-by-50-kb bin at the center of the matrix was measured to generate the CSS for the interaction of interest. To measure the percent change in CSS between timepoints in Liquid Hi-C, the average & standard deviation of CSS for 3 biological replicates was measured per timepoint per interaction (active OR-GI vs. inactive ORs-GI). The percent change in CSS per replicate per timepoint was measured against the average CSS at 0-min of Liquid Hi-C (control).

Loss of Structure in Liquid Hi-C
Loss of structure calculation was performed as described in Belaghzal et al. 2021[26] with the following modifications. To examine increasing loss of structure with increased lengths of pre-digestion, normalized contacts made by a 50-kb bin to a 3-Mb radius in *cis* were divided by the total number of contacts made across the chromosome, generating the loss of structure score (LOS) for that bin. Successful Liquid Hi-C was verified by observing higher levels of LOS for compartment A vs. compartment B chromatin, that increased with longer pre-digestion. Compartment profiles were generated by performing eigendecomposition on 50-kb binned cooler files with cooltools.

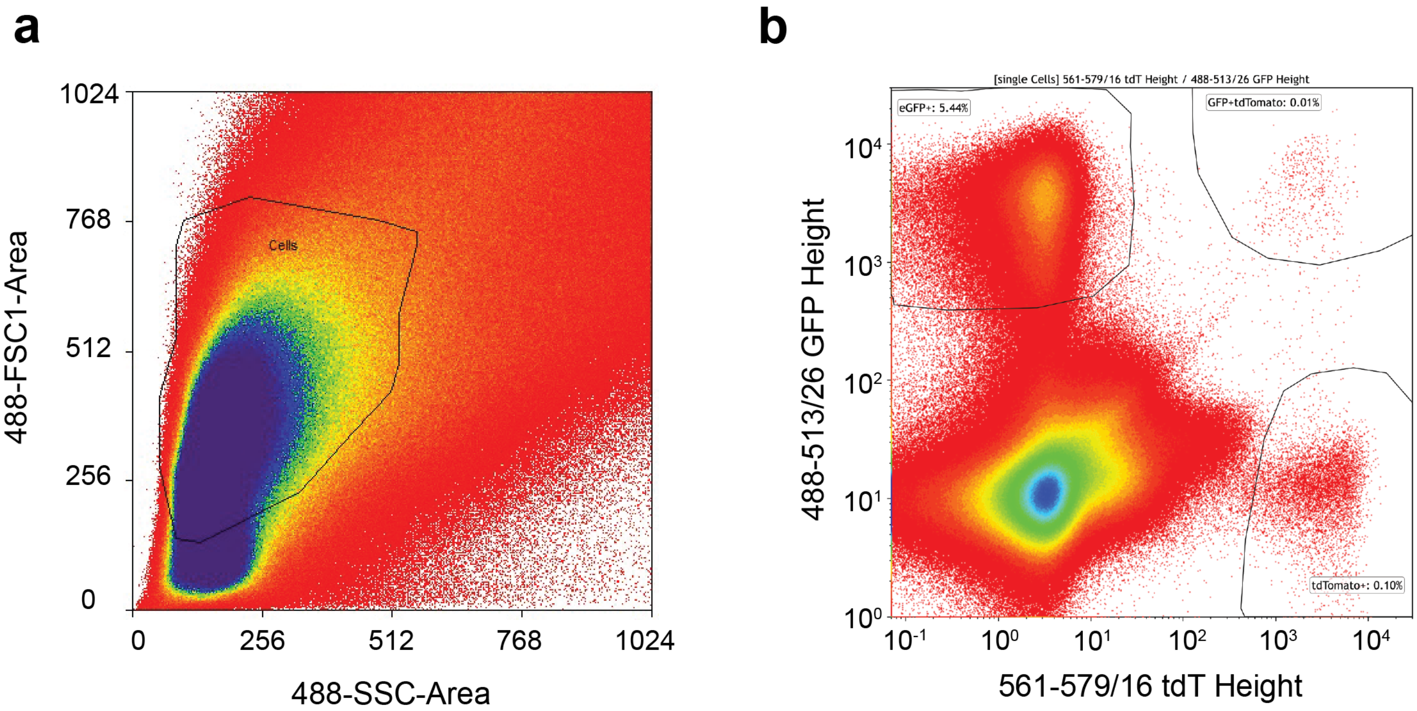

Supplemental Information Figure 1 (**a-b**) The FACS plot in Supplemental Information Figure 1 is a representative FACS plot illustrating our gating strategy for purifying GFP+/tdT-, GFP-/tdT+, and GFP+/tdT+ cells. This gating strategy was also used to isolate GFP+ cells in on our other experiments from other mouse lines. (a) Briefly, for this plot, MOE were dissected from OMPtTA>tetOP2iGFP, mor28iCre>tdTom fl/+, mice and dissociated to obtain a single cell suspension of the MOE which was suspended in sort solution of 2% FBS/1xPBS, 4 mM MgCl2, and 1:1000 DAPI. 300 uL of sort solution was used per OE. FSC/SSC gates are set to eliminate small debris as well as larger and more granular cells that are not olfactory neurons. Doublet discrimination is performed by plotting SSC Height vs. SSC area to eliminate events with larger area, indicating they are doublets. This is also done by comparing SSC Height vs. SSC width to eliminate events with greater width which would represent doublets. Gating is also performed to eliminate DAPI positive cells. (b) Both GFP+ and tdT+ cells are three decades brighter than negative cells and are clear cell populations which can be easily defined (GFP = 5.44%, tdT+ = 0.1%, GFP+/tdT+ = 0.01%).
