# Supplementary material for: RNA-mediated symmetry breaking enables singular olfactory receptor choice
Source: Nature. 2023 Dec 20;625(7993):181–8. doi: 10.1038/s41586-023-06845-4 (PMC10765522; doi:10.1038/s41586-023-06845-4)
Supplement: Supplementary file 2 — Reporting Summary [file 41586_2023_6845_MOESM2_ESM.pdf]

nature portfolio

Corresponding author(s): Stavros Lomvardas

Last updated by author(s): September 29 2023

Reporting Summary

Nature Portfolio wishes to improve the reproducibility of the work that we publish. This form provides structure for consistency and transparency in reporting. For further information on Nature Portfolio policies, see our Editorial Policies and the Editorial Policy Checklist.

Statistics

For all statistical analyses, confirm that the following items are present in the figure legend, table legend, main text, or Methods section.

n/a	Confirmed	
☐	☒	The exact sample size (*n*) for each experimental group/condition, given as a discrete number and unit of measurement
☐	☒	A statement on whether measurements were taken from distinct samples or whether the same sample was measured repeatedly
☐	☒	The statistical test(s) used AND whether they are one- or two-sided<br>*Only common tests should be described solely by name; describe more complex techniques in the Methods section.*
☒	☐	A description of all covariates tested
☒	☐	A description of any assumptions or corrections, such as tests of normality and adjustment for multiple comparisons
☐	☒	A full description of the statistical parameters including central tendency (e.g. means) or other basic estimates (e.g. regression coefficient) AND variation (e.g. standard deviation) or associated estimates of uncertainty (e.g. confidence intervals)
☐	☒	For null hypothesis testing, the test statistic (e.g. *F*, *t*, *r*) with confidence intervals, effect sizes, degrees of freedom and *P* value noted<br>*Give P values as exact values whenever suitable.*
☒	☐	For Bayesian analysis, information on the choice of priors and Markov chain Monte Carlo settings
☐	☒	For hierarchical and complex designs, identification of the appropriate level for tests and full reporting of outcomes
☒	☐	Estimates of effect sizes (e.g. Cohen's *d*, Pearson's *r*), indicating how they were calculated

Our web collection on statistics for biologists contains articles on many of the points above.

Software and code

Policy information about availability of computer code

Data collection	Zeiss Zen2012 SP1 (v8.1.9.484) was used for capturing confocal images
Data analysis	BWA 0.7.17<br>hickit r291<br>k8-Linux K8: 0.2.5-r80<br>Bedtools2 v2.26.0<br>conda 4.11.0<br>Python v2.7.13<br>PyMOL(TM) 2.5.3<br>dip-c (https://github.com/tanlongzhi/dip-c) (81dc83ea824c3503f835ba7b0d333163c46b9aca, Jan 9, 2019)<br>cellranger-arc-2.0.1<br>macs2 2.2.7.1<br>Signac 1.6.0<br>Seurat 4.1.0<br>R 4.1.3<br>monocle3 1.0.0<br>java 8<br>nextflow version 19.07.0.5106<br>Docker version 19.03.2, build 6a30dfc<br>distiller-nf 0.3.3<br>cooler, version 0.8.10

Integrated Genome Browser 9.0.0
CutAdapt v1.17
Bowtie2 v2.3.2
Samtools v1.4.1
STAR v2.5.3a
ImageJ 2.0.0
Picard 1.137
DeSeq2 1.24.0

Scripts for all data analysis were written in R and is available under the following github repository (https://github.com/arielpourmorady/Pourmorady_etal.git)

For manuscripts utilizing custom algorithms or software that are central to the research but not yet described in published literature, software must be made available to editors and reviewers. We strongly encourage code deposition in a community repository (e.g. GitHub). See the Nature Portfolio guidelines for submitting code & software for further information.

Data

Policy information about availability of data

All manuscripts must include a data availability statement. This statement should provide the following information, where applicable:

- Accession codes, unique identifiers, or web links for publicly available datasets
- A description of any restrictions on data availability
- For clinical datasets or third party data, please ensure that the statement adheres to our policy

Data that support the findings of this study are deposited in a GEO superseries with the following accession number: GSE230380. Dip-C data from previously published work from our lab were used for Fig. 2b and Fig. S5b (GSE158730). Dip-C data from Tan et al. were used to render PCAs on single-cell chromatin compartments in Fig. S8e-f (GSE121791). Previously published Hi-C data from our lab were used for Fig. S8a-f, and are publicly available at https://data.4dnucleome.org/ under the following accession numbers: 4DNESH4UTRNL, 4DNESNYBDSLY, 4DNES54YB6TQ, 4DNESRE7AK5U, 4DNES425UDGS and 4DNESEPDL6KY. Genome assembly for mm10 genome which was used for deep-sequencing read alignment can be found at https://www.ncbi.nlm.nih.gov/datasets/genome/GCF_000001635.20/.

Human research participants

Policy information about studies involving human research participants and Sex and Gender in Research.

Reporting on sex and gender	N/A
Population characteristics	N/A
Recruitment	N/A
Ethics oversight	N/A

Note that full information on the approval of the study protocol must also be provided in the manuscript.

Field-specific reporting

Please select the one below that is the best fit for your research. If you are not sure, read the appropriate sections before making your selection.

☒ Life sciences ☐ Behavioural & social sciences ☐ Ecological, evolutionary & environmental sciences

For a reference copy of the document with all sections, see nature.com/documents/nr-reporting-summary-flat.pdf

Life sciences study design

All studies must disclose on these points even when the disclosure is negative.

Sample size

Dip-C was performed on sorted cells pooled from at least three mice and performed independently on gg8tTA>tetO-P2 and mor28iGFP mouse lines. Dip-C sample size was based off Tan et al. 2019, where authors also examined Dip-C models and contacts of nuclei in 409 cells which represented a mixed population of cell types of the MOE including mOSNs. Since we performed Dip-C on a pure population of sorted mOSNs from the MOE, we targeted our sample size to 192 cells and generated 161 high quality cells. The multiome was generated from one mouse and reproduced independently from a second mouse (shown is supplement). For bulk high throughput sequencing experiments, a sample size of two to three independent biological replicates was selected. For Hi-C experiments (including HiChIP and Liquid Hi-C), at least two independent samples were generated as per 4DNucleome guidelines (https://drive.google.com/file/d/1-NEldtpuDuYXcbWngETltNPCRv0RZlIK/view). Furthermore, for HiChIP and ATAC-seq experiments, at least two independent samples were generated as per ENCODE guidelines (https://doi.org/10.1101/gr.136184.111). For RNA-Seq, 2-3 biological replicates were selected because multiple statistical approaches have been developed to allow identification of significantly changed loci or genes from two biological replicates of high throughput sequencing data (e.g. edgeR, DEseq2). These approaches work by using the large number of genes/loci measured to

	analyze and model the dispersion and variance within and between replicates, thereby allowing the identification of genes/loci with significant differences between conditions. Wherever possible, additional biological replicates were included.
Data exclusions	For the multiome, cells was quality controlled by retaining cells with a TSS enrichment > 1, nucleosome signal < 2, ATAC counts between 1,000 and 10,000, and RNA counts between 1,000 and 25,000. For scATAC data from the multiome, peaks within ENCODE black-listed regions (https://sites.google.com/site/anshulkundaje/projects/blacklists) were excluded from analysis. This exclusion was pre-established. For Dip-C, we excluded cells that had contacts < ~400,000 contacts, cells that had a low contact-to-read ratio, and cells that had high variability in 3D structure across computational replicates.
Replication	At least two independent biological replicates were performed for each experiment. Replicate experiments yielded the same results.
Randomization	No experiments were performed with live animals. For the purpose of purifying primary cells, animals of similar age were grouped by genotype and sorted together.
Blinding	Animals were used as a source of tissue and primary cells, so knowledge of genotype was required for proper handling and cell sorting. Every genotype analyzed contained multiple transgenic alleles, consequently only a small fraction of mice had the experimental and control genotypes; therefore, knowledge of genotype was essential for deciding which animals to use for FAC-sorting and downstream experiments. Performing the reported experiments in a blind fashion would increase the cost of the analysis by 10-fold, and would result in unnecessary preservation of mice that should be euthanized. Finally, because our analysis was performed in transgenic mice, blinding is not possible because the genotypes become apparent in the analysis itself.

Reporting for specific materials, systems and methods

We require information from authors about some types of materials, experimental systems and methods used in many studies. Here, indicate whether each material, system or method listed is relevant to your study. If you are not sure if a list item applies to your research, read the appropriate section before selecting a response.

Materials & experimental systems

n/a	Involved in the study
☐	☒ Antibodies
☒	☐ Eukaryotic cell lines
☒	☐ Palaeontology and archaeology
☐	☒ Animals and other organisms
☒	☐ Clinical data
☒	☐ Dual use research of concern

Methods

n/a	Involved in the study
☒	☐ ChIP-seq
☐	☒ Flow cytometry
☒	☐ MRI-based neuroimaging

Antibodies

Antibodies used	GFP (chicken anti-GFP ab13970)<br>M71 (Lomvardas et al. 2006, Gilad Barnea Brown University)<br>P2 (Olfr17 antibody were raised in guinea pig, Gilad Barnea Brown University)<br>LacZ (abcam ab4761)<br>anti-chick IgG conjugated to Alexa-488 (Jackson ImmunoResearch, Code 103-434-155, RRID: AB_2337390 polyclonal).<br>anti-guinea pig IgG conjugated to Cy3 (Jackson ImmunoResearch, Code 106-165-003, RRID: AB_2337423 polyclonal).<br>H3K27ac antibody (Abcam GR323193701)
Validation	LacZ (abcam ab4761) - Manufacturer states that antibody is validated for immunofluorescenc<br>GFP (chicken anti-GFP ab13970) - Manufacturer states that antibody is validated for immunofluorescence<br>anti-guinea pig IgG conjugated to Cy3 - Manufacturer states that antibody is validated for immunofluorescence<br>anti-chick IgG conjugated to Alexa-488 - Manufacturer states that antibody is validated for immunofluorescence<br>H3K27ac antibody (Abcam GR323193701) - Manufacturer states that antibody is validated for immunofluorescence<br>M71 antibody - Validated in Lomvardas et al. 2006 and in this study by positive staining in the cilia of neurons expressing the tetO-M71-LacZ transgene and negative staining in tetO-M71(KO)-IRES-GFP mice.<br>P2 antibody - Validated in this study by positive staining in the cilia of neurons expressing the tetO-P2iresGFP mutant gene and negative staining in tetO-P2(KO)iresGFP.

Animals and other research organisms

Policy information about studies involving animals; ARRIVE guidelines recommended for reporting animal research, and Sex and Gender in Research

Laboratory animals	The mice are housed in individually ventilated cages (IVC), and the rooms are maintained at 72F and 30%RH. The light cycle is 12:12 (lights on at 7:00am off at 7:00pm). Mice are fed irradiated PicoLab Rodent Diet 20, and are housed in cages with irradiated corn cob bedding. This study used several mouse lines (mus musculus) on mixed C57BL/6J and 129 backgrounds. Experimental mice were generated by crossing mice from the following lines together in different combinations. All experiments were performed on male and female adult mice between 5-12 weeks of age.

Experimental genotypes were:

Gng8(gg8)-tTA - Male and female adult mice were used
OMPitTA - Male and female adult mice were used
tetO-P2-IRES-GFP - Male and female adult mice were used
tetO-P2(KO)-IRES-GFP - Male and female adult mice were used
tetO-M71-LacZ - Male and female adult mice were used
tetO-M71(KO)-IRES-GFP - Male and female adult mice were used
tetO-GFP - Male and female adult mice were used
Olfr1507-ires-GFP - Male and female adult mice were used
CAST/EiJ - Male and female adult mice were used
Atf5irep - Male and female adult mice were used
OMP-ires-GFP - Male and female adult mice were used
Ngn1-GFP - Male and female mice were used a 1-2 weeks of age.
mor28-ires-cre - Male and female adult mice were used
Rosa26(LSL-tdTomato/+) - Male and female adult mice were used

Data published in Horta et al. 2019 were generated from the following mice and used in this paper:

Krt5Cre;R26R-tdtomato-GFP - Male and female adult mice were used
Olfr17-ires-GFP - Male and female adult mice were used

Wild animals	None
Reporting on sex	All experiments were performed on both sexes and findings apply to both sexes.
Field-collected samples	None
Ethics oversight	IACUC - Columbia University

Note that full information on the approval of the study protocol must also be provided in the manuscript.

Flow Cytometry

Plots

Confirm that:

☒ The axis labels state the marker and fluorochrome used (e.g. CD4-FITC).

☒ The axis scales are clearly visible. Include numbers along axes only for bottom left plot of group (a 'group' is an analysis of identical markers).

☒ All plots are contour plots with outliers or pseudocolor plots.

☒ A numerical value for number of cells or percentage (with statistics) is provided.

Methodology

Sample preparation	The FACS plot in Supplemental Figure 9a is a representative FACS plot illustrating our gating strategy for purifying GFP+/tdT-, GFP-/tdT+, and GFP+/tdT+ cells. This gating strategy was also used to isolate GFP+ cells in on our other experiments from other mouse lines. Briefly, for this plot, MOE were dissected from OMPtTA>tetOP2iGFP, mor28iCre>tdTom fl/+, mice and dissociated to obtain a single cell suspension of the MOE which was suspended in sort solution of 2% FBS/1xPBS, 4 mM MgCl2, and 1:1000 DAPI. 300 uL of sort solution was used per OE.
Instrument	Beckman Coulter Low Flow Astrios EQ
Software	Acquisition Software: Summit v3.2.1
Cell population abundance	For OMPtTA>tetOP2iGFP, mor28iCre>tdTom fl/+, experiments: GFP+ 5.44%, tdT+ 0.1%, and GFP+/tdT+ 0.01%.
Gating strategy	FSC/SSC gates are set to eliminate small debris as well as larger and more granular cells that are not olfactory neurons. Doublet discrimination is performed by plotting SSC Height vs. SSC area to eliminate events with larger area, indicating they are doublets. This is also done by comparing SSC Height vs. SSC width to eliminate events with greater width which would represent doublets. Gating is also performed to eliminate DAPI positive cells. Both GFP+ and tdT+ cells are three decades brighter than negative cells and are clear cell populations which can be easily defined.

☒ Tick this box to confirm that a figure exemplifying the gating strategy is provided in the Supplementary Information.
